# Metabolic Imaging as Future Technology and Innovation in Brain-Tumour Surgery: A Systematic Review

**DOI:** 10.3390/curroncol32110597

**Published:** 2025-10-24

**Authors:** Thomas Kapapa, Ralph König, Jan Coburger, Benjamin Mayer, Kornelia Kreiser, Volker Rasche

**Affiliations:** 1 Department for Neurosurgery, Ulm University Hospital, Albert-Einstein-Allee 23, 89081 Ulm, Germany; 2 Department for Neurosurgery, Bezirkskrankenhaus Günzburg, Ulm University, Lindenallee 2, 89312 Günzburg, Germany; 3 Institute for Epidemiology and Medical Biometry, Ulm University, Schwabstr. 13, 89075 Ulm, Germany; 4 Rehabilitationskrankenhaus Ulm, Ulm University Hospital, Oberer Eselsberg 45, 89081 Ulm, Germany; 5 Center for Translational Imaging, Ulm University, Albert-Einstein-Allee 23, 89081 Ulm, Germany

**Keywords:** glioma, glioblastoma, MRI, intraoperative imaging, gross total resection, save surgery, brain tumour

## Abstract

**Background:** Standard imaging in neurosurgery often fails to visualize infiltrative tumor regions that extend beyond contrast enhancement. Metabolic imaging using hyperpolarized 13C-MRI may offer new intraoperative insights into tumor biology. **Objective:** To systematically assess the clinical and technical evidence on hyperpolarized MRI for metabolic tumour characterization in patients with malignant brain tumors. **Eligibility criteria:** We included original human studies reporting on hyperpolarized 13C-MRI for perioperative and diagnostic use in brain tumor patients. Reviews, animal studies, and technical-only reports were excluded. **Information sources:** Searches were conducted in PubMed, Embase, and Web of Science on 26 December 2024. **Risk of bias:** Methodological quality was assessed using the QUADAS-2 tool. **Synthesis of results:** A qualitative synthesis was performed, and where feasible, random-effects meta-analysis was used to calculate standardized mean differences (SMDs) and heterogeneity statistics. **Results:** Three studies (n = 15 patients) met inclusion criteria. The bicarbonate-to-pyruvate ratio showed a significant difference between tumor and non-tumour brain (SMD = 1.34, *p* = 0.002), whereas pyruvate-to-lactate ratio (kPL) values showed minimal difference (SMD = 0.06, *p* = 0.730). Asmall effect was observed for kPL between tumor and normal-appearing white matter (SMD = –0.33). One study provided qualitative data only. Overall heterogeneity was high (I^2^ = 69.4%). **Limitations:** Limitations include small sample sizes, heterogeneous methodologies, and limited availability of patient-level data. **Interpretation:** Hyperpolarized 13C-MRI shows metabolic differentiation between tumor and healthy tissue in certain parameters, especially bicarbonate metabolism. While promising, the technology requires further clinical validation before routine intraoperative application.

## Simple Summary

Malignant brain tumors are known to undergo profound metabolic changes that drive their growth and resistance to therapy. Understanding these metabolic shifts in living patients remains a major challenge in oncology. This study explores the use of hyperpolarized magnetic resonance imaging, a novel method that enables non-invasive, real-time measurement of key metabolic pathways directly in the tumour. By focusing on conversion of pyruvate into lactate or bicarbonate, this approach may provide insights into tumour aggressiveness and treatment response. Our findings suggest that mapping tumour metabolism in this way could help (neuro-)oncologists and neurosurgeons to improve diagnostic precision, guide targeted and more precise resection therapy, and monitor disease progression more effectively in patients with intrinsic brain cancer.

## 1. Introduction

In cases of primary or intrinsic brain tumours, imaging assumes a particularly crucial role [1,2,3]. Primary brain tumours, such as gliomas, are notorious for their infiltrative growth patterns, often extending beyond the boundaries visualised on conventional imaging [3,4]. This presents a significant challenge for neurosurgeons aiming to achieve a gross total resection (GTR), as residual tumour cells may compromise long-term patient outcomes [5,6,7]. Numerous international studies have highlighted the importance of GTR in improving patient survival and quality of life [8,9,10,11,12,13,14,15,16,17,18,19]. Several landmark studies have established that the extent of resection is a key prognostic factor [11,20,21,22,23,24,25]. Achieving at least a certain percentage of tumour volume removal—often cited as more than 90% or even closer to 100% of the contrast-enhancing tumour—improves survival and postpones the need for adjuvant therapies [22,24,26,27,28,29,30,31,32].

Contrast-enhanced magnetic resonance imaging (MRI), currently considered the gold standard for preoperative planning, relies on the disruption of the blood–brain barrier (BBB) to delineate tumour margins [33,34,35,36]. However, both high- and low-grade gliomas frequently contain non-enhancing regions, resulting in underestimation of the tumour’s true extent [34,37,38]. This discrepancy creates a dilemma in current clinical practice: neurosurgeons strive to maximise resection for better oncological outcomes but must rely on imaging that inherently underrepresents tumour infiltration [22,26]. In summary, the primary limitation remains that the imaging contrast used to define tumour boundaries is biochemical rather than metabolic [39,40]. Some tumours infiltrate the surrounding normal-appearing brain tissue extensively, and the surgeon’s ability to completely remove all tumour cells without neurological compromise is constrained by the limited imaging information available. Residual microscopic disease often persists [7,22,41,42,43]. The clinical and scientific gap between radiologic GTR—defined by the absence of contrast-enhancing tissue—and the actual microscopic infiltration of tumour cells remains a fundamental challenge in neuro-oncological surgery [44,45,46,47].

Thus far, no widely adopted intraoperative method reliably delineates the true metabolic extent of a tumour in real time [30,31,48,49,50,51,52,53,54,55,56,57,58,59,60,61,62]. The implications of this imaging limitation extend beyond the surgical operating room. Postoperative MRI, obtained within hours or days after surgery, often shows residual tumour that was not visible intraoperatively [7,63]. This finding underscores the continuing need for improved intraoperative imaging techniques that can assist the surgeon to identify and resect tumour tissue more completely while sparing normal, eloquent brain areas.

Novel approaches such as intraoperative metabolic imaging, Raman spectroscopy, and desorption electrospray ionisation mass spectrometry (DESI-MS) are being investigated to address this gap and could pave the way for real-time metabolic tumour mapping during surgery [7,64,65,66,67,68]. One of the most innovative and promising directions in neurosurgical imaging lies in metabolic imaging techniques that can be integrated intraoperatively. Among these advances, hyperpolarized magnetic resonance imaging (hMRI) has emerged as a transformative technology [69,70,71,72,73,74,75,76,77,78,79]. This technique enhances signal intensities of MR-visible nuclei, such as carbon-13, by several orders of magnitude, enabling real-time visualization of metabolic processes with unprecedented clarity [80,81,82,83,84,85,86,87,88,89,90,91,92,93]. hMRI has demonstrated its ability to probe dynamic metabolic activity, particularly the conversion of pyruvate into lactate and bicarbonate, which are key indicators of glycolytic activity and mitochondrial function [88,90,92,94]. The technique’s ability to monitor metabolic fluxes in vivo has been validated through its application in gliomas, where hyperpolarized [1-13C]pyruvate has provided detailed insights into the Warburg effect—tumour cells favour aerobic glycolysis even when enough oxygen is available—and tumour heterogeneity [82,85,88,95,96,97]. Tracking pyruvate-to-lactate conversion has proven to be a sensitive marker for identifying tumour activity and guiding resections [88,98]. Furthermore, evidence suggests that hyperpolarized [2-13C]pyruvate offers additional insights into tumour metabolism [76,82,89,90,91,99]. Recent advances in hMRI have also extended its applications to cerebral perfusion [82,100,101,102,103,104,105,106].

Moreover, hMRI enables the detection of residual tumour activity and supports the development of personalized adjuvant therapies [80,91,94]. Despite its transformative potential, hMRI faces challenges in clinical translation. The transient nature of hyperpolarization limits imaging timeframes, and the technique requires specialized infrastructure and expertise [72,107]. However, ongoing advancements in portable hyperpolarization systems and novel protocols hold promise for broader adoption in clinical practice [72,82,95,107,108]. By bridging the gap between structural and metabolic imaging, hMRI could represent a paradigm shift in neurosurgical imaging [82,109].

Importantly, achieving a genuine benefit from metabolic imaging advances like hMRI requires not only technological innovation but also integration with evolving neuropathological classifications like the 2021 WHO central nervous system tumour classification [110,111,112]. However, these classifications often rely on time-intensive molecular techniques, such as next-generation sequencing and methylation profiling, which can delay the initiation of adjuvant treatments and have limited utility for intraoperative decision-making [112]. While advances in artificial intelligence (AI) have begun to address this challenge by improving the speed and accuracy of molecular characterisation, their integration into routine neurosurgical workflows remains limited [113]. Current intraoperative imaging modalities provide little guidance regarding the metabolic or molecular nature of the tissue at the tumour margin. hMRI, by contrast, offers a metabolic perspective that might correlate more closely with the underlying tumour biology and aggressiveness [114]. In the future, this could lead to an era where surgeons not only localize the lesion precisely but also tailor their resection to the lesion’s metabolic and genetic profile, ultimately improving patient survival and quality of life.

This article will introduce the concept of hMRI in neurosurgery and explore the chronological development of imaging and technical innovations in neurosurgery, and review the role hMRI versus various intraoperative imaging modalities in guiding tumour resections. We will discuss how this cutting-edge technology may help overcome the limitations of traditional contrast-based imaging, thereby heralding a paradigm shift in neurosurgical and neuro-oncological practice. A systematic review approach was chosen to summarize the amount of information on this topic.

## 2. Materials and Methods

This systematic review was conducted according to PRISMA guidelines (PROSPERO ID: 1161380) [115]. It was combined with a narrative review to demonstrate the historical and technological pathway for preoperative, intraoperative and postoperative brain tumour imaging. Where appropriate, results were combined with random-effects meta-analysis. As this is an analysis of previously published data, no ethical approval was required. This study was not funded.

### 2.1. Inclusion and Exclusion Criteria

Studies were included if they met the following criteria:

- Focused on the application of hyperpolarized MRI in patients undergoing neurosurgical procedures or in the management of primary brain tumours.
- Reported quantitative or qualitative outcomes related to imaging.
- Published as peer-reviewed original research articles.

Exclusion criteria were:

- Reviews, case reports, editorials, or non-peer-reviewed studies.
- Studies involving animal models or in vitro experiments without patient data.
- Articles published in languages other than English.

### 2.2. Study Selection Process

**Figure 1:**
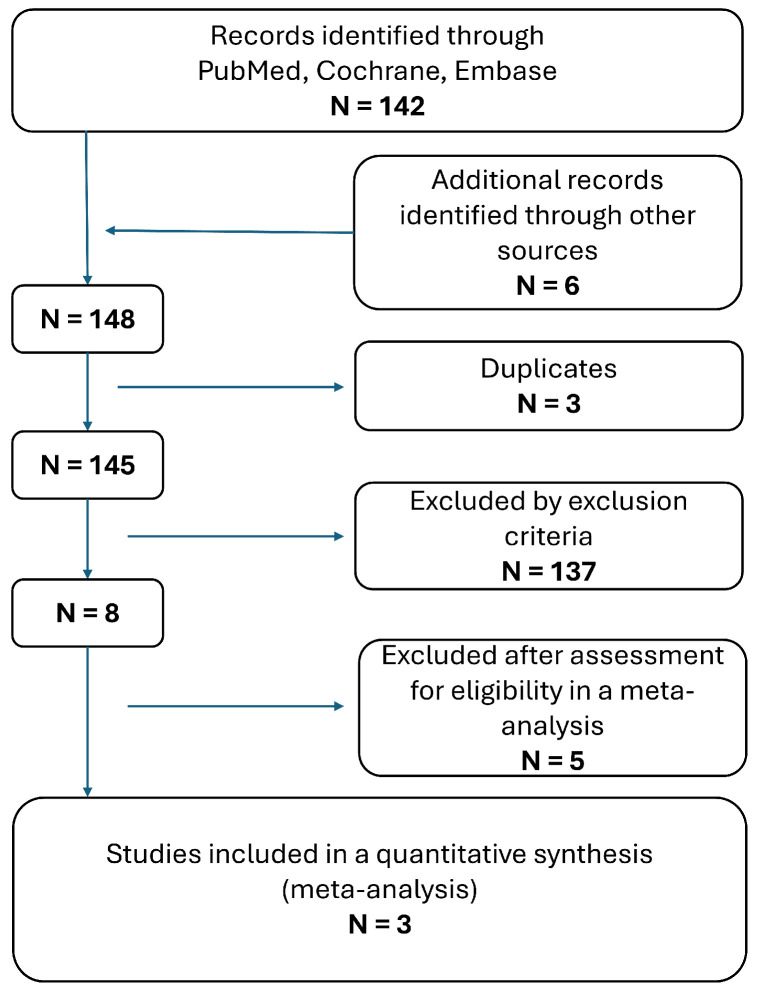
Literature selection process following the PRISMA guidelines.

The selection process followed PRISMA guidelines [115]. Two reviewers independently screened titles and abstracts for relevance. Full-text articles of potentially eligible studies were retrieved and assessed for inclusion also by the two independent reviewers. Discrepancies in study selection were resolved through discussion or consultation with a third reviewer. The selection process was documented and is summarized in the PRISMA flow diagram (Figure 1).

The literature search for the systematic review was conducted in the PubMed, Embase and Cochrane Library databases on 26 December 2024. The search strategy included terms such as ‘hyperpolarised MRI’ (“Hyperpolarized Magnetic Resonance Imaging”[Mesh] OR “hyperpolarized MRI” OR “hyperpolarized magnetic resonance” OR “hyperpolarized 13C MRI” OR “hyperpolarized imaging”), ‘brain tumour’ (“Brain Tumors”[Mesh] OR “brain tumor” OR “glioma” OR “astrocytoma” OR “oligodendroglioma” OR “glioblastoma” OR “intracranial neoplasm” OR “brain neoplasm”) and ‘neurosurgery’(“neurosurgery” OR “neurosurgical procedure” OR “brain surgery”). The terms “preoperative”, “intraoperative”, “postoperative”, “therapy”, “treatment monitoring”, or “surgical planning” for the temporal categorisation of the application within the therapy have also been added. Boolean operators (AND, OR) and field-specific tags (e.g., [Title/Abstract]) were utilized to refine the search. Filters for English-language publications and studies published between 2010 and 2024 were applied to ensure relevance and currency. Only peer-reviewed articles were included.

### 2.3. Data Extraction

Data from included studies were extracted independently by two reviewers using a standardized data extraction form. Extracted variables included study design, sample size, patient demographics, imaging protocols, and primary outcomes related to hyperpolarized MRI. Disagreements during data extraction were resolved by consensus.

### 2.4. Quality Assessment

The methodological quality of the included studies was systematically evaluated using the QUADAS-2 (Quality Assessment of Diagnostic Accuracy Studies) tool. This tool assesses the risk of bias and applicability concerns in four key domains:

**Patient Selection**: Evaluates whether the included patient populations were representative of the clinical scenario.

**Index Test**: Examines whether the hyperpolarized MRI protocols were described clearly and applied consistently.

**Reference Standard**: Assesses the adequacy and reliability of the reference standards used to validate MRI findings.

**Flow and Timing**: Ensures that all patients underwent the index test and reference standard in an appropriate timeframe without exclusions that could bias the results.

Each study was evaluated independently by two reviewers. For each domain, risk of bias and applicability concerns were rated as **low**, **high**, or **unclear** using the QUADAS-2 signalling questions framework. Discrepancies were resolved through consensus or consultation with a third reviewer.

The following scoring formula was used for each domain:

Domain Score = Low Bias: if all signalling questions are answered ‘yes’;                                                 High Bias: if one or more signalling questions are answered ‘no’;                          Unclear Bias: if insufficient information is provided.

An aggregate score for each study was calculated to provide an overall quality rating. Scores across studies were presented as proportions for descriptive comparison. The results of the quality assessment are summarized in Table S1.

### 2.5. Assessment of Heterogeneity and Statistical Analysis

Heterogeneity refers to the degree of variation in study outcomes due to clinical, methodological, or statistical differences. Evaluating heterogeneity is essential to determine whether pooling results in a meta-analysis is appropriate. Heterogeneity among the included studies was evaluated using Cochran’s Q-test for heterogeneity, the I^2^ statistic, and the Tau^2^ statistic. Cochran’s Q statistic tests whether the observed variation in effect sizes is greater than expected by chance. The formula is given by: Q = Σw_i_ (y_i_ − ȳ)^2^. Where: w_i_ = weight of study i (1/variance), y_i_ = effect size of study I, ȳ = pooled effect size (weighted mean), k = number of studies. The I^2^ statistic quantifies the proportion of variability due to heterogeneity rather than chance. It is calculated as: I^2^ = max(0, (Q − df)/Q) × 100. In this equation, Q = Cochran’s Q, and df = degrees of freedom (k − 1). Tau^2^ estimates the between-study variance and is used in random-effects models. It is calculated as: Tau^2^ = (Q − df)/Σw_i_, if Q > df; otherwise, Tau^2^ = 0. These measures assess the variability in effect sizes across studies and guide the choice of the appropriate meta-analytical model in a way that a random effects model is suggested to apply in case of I^2^ > 50%. Calculations were performed by SPSS-Software Version 29 (IBM SPSS Statistics, Armonk, NY, USA), Microsoft Excel Software (Microsoft, Redmond, WA, USA), and GraphPad Prism Version 10.4.1 (Boston, MA, USA).

## 3. Results

A total of three relevant studies were identified through a systematic literature search that investigated the application of hMRI in primary brain tumours surgery [69,98,116]. All studies employing hMRI in glioma and glioblastoma patients were included in this meta-analysis. Despite methodological similarities in imaging protocols, the study designs, result presentations, patient characteristics, and outcome measures varied across cohorts (Table 1). All employed 3 Tesla MRI systems equipped with specialized ^13^C coils. Control regions were defined either as contralateral normal-appearing white matter (NAWM) or unaffected hemispheric tissue (normal appearing brain parenchyma, NABP). Despite differences in cohort size and outcome metrics, each study applied hMRI in a patient-specific clinical setting prior to therapeutic intervention, supporting the modality’s feasibility and reproducibility in neurosurgery.

### 3.1. Application of Hyperpolarized MRI in Neurosurgery

Hyperpolarized MRI was applied across all three included studies in patients diagnosed with primary brain tumours, specifically gliomas and glioblastomas. Although none of the studies reported explicit intraoperative or surgical navigation applications, the imaging was consistently performed in vivo during the preoperative phase, thereby providing functional metabolic information potentially valuable for neurosurgical decision-making.

In the study by Autry et al., hyperpolarized ^13^C pyruvate MRI was performed in five patients with glioma [116]. Imaging revealed elevated conversion rates of pyruvate to lactate (k_PL) and bicarbonate (k_PB) within tumour regions as compared to NAWM, indicating the feasibility of detecting altered tumour metabolism prior to surgery. These findings suggest that hMRI could contribute to the metabolic characterization of tumour tissue beyond anatomical, contrast medium enhanced MRI.

Chen et al. conducted hMRI in three patients with histologically confirmed glioblastoma [98]. The study demonstrated detectable and spatially localized ^13^C lactate signals in contrast-enhancing tumour regions, with minimal signal in contralateral white matter. While no quantitative outcome measures were reported beyond relative signal presence, the spatial correlation between metabolic activity and anatomical lesion morphology points toward potential clinical applications in tumour delineation and planning.

In the study by Zaccagna et al., seven patients with glioma underwent hyperpolarized ^13^C pyruvate MRI, and the authors specifically quantified the lactate-to-bicarbonate signal ratio within tumours and contralateral brain tissue [69]. The significantly elevated lactate-to-bicarbonate ratio in tumour tissue (mean = 0.1043 vs. 0.0571; *p* = 0.002) suggests a distinct metabolic fingerprint of gliomas. Although the authors did not implement the imaging in a neurosurgical workflow, the ability to non-invasively characterize aerobic glycolysis in vivo presents a clear rationale for clinical translation, especially in the context of preoperative mapping or biopsy targeting.

To enable comparison across studies, standardized mean differences (SMDs) were calculated for metabolic imaging parameters reported in Zaccagna and Autry. In the study by Zaccagna et al., the lactate-to-bicarbonate ratio showed significant difference between tumor and contralateral normal-appearing brain parenchyma (SMD = 1.34, SE = 0.600, 95% CI: [−2.51, 0.16], *p* = 0.002). In contrast, the kPL values (pyruvate-to-lactate conversion rate) in the same study revealed only a minor difference between tumor and non-tumor regions (SMD = 0.06, SE = 0.535, *p* = 0.730). Autry et al. reported kPL values across five patients, with a small difference between tumor tissue and normal-appearing white matter (SMD = –0.33, SE = 0.638, 95% CI: [–1.58, 0.92]). Data from Chen et al. did not allow for numerical synthesis due to absence of group-level mean values, but the authors reported signal enhancement in tumor regions without quantifying effect sizes (Table 2).

Taken together, the included studies demonstrate the feasibility and metabolic sensitivity of hMRI in glioma patients. While direct intraoperative use was not investigated, the results underscore the modality’s potential as a non-invasive adjunct to standard imaging in neurosurgical oncology.

### 3.2. Quality Assessment

The methodological quality of the included studies was evaluated using the QUADAS-2 tool. A summary of the assessments is provided in Table S1. All three studies were judged to have a low risk of bias in patient selection, with clearly defined inclusion criteria and no indication of inappropriate exclusions. Autry et al. was rated as low risk in three domains. The reference standard was rated as unclear due to the absence of histopathological validation to confirm metabolic interpretations derived from hMRI. Although clinical context supported lesion localization, no formal gold standard was applied per region of interest [116]. Chen et al. received an unclear rating in the index test domain, as no description was provided regarding blinding of image interpreters or use of predefined diagnostic thresholds [98]. The flow and timing domain was also rated as unclear, given the lack of reporting on whether all enrolled patients received both index and reference tests within the same diagnostic pathway. Zaccagna et al. demonstrated overall low risk, though unclear risk was assigned in the reference standard domain due to the absence of lesion-specific histological correlation [69]. Blinding of assessors was not described.

Overall, the studies demonstrated acceptable methodological rigor. Minor limitations related to documentation of blinding and reference validation were common and contributed to “unclear” ratings in some domains. None of the included studies demonstrated evidence of systematic high risk of bias.

### 3.3. Heterogeneity of the Studies

Heterogeneity analysis was performed using data from the two studies that reported extractable quantitative outcome measures suitable for meta-analytic synthesis: Autry et al. [116] and Zaccagna et al. [69]. Both studies provided figures to calculate or directly withdraw standardized mean differences (SMDs) with reference to tumour versus control regions based on hMRI metrics.

The resulting heterogeneity was according to the different study concept results (Lactate/Bicarbonate ratio in GBM and NABP versus kPL in T2L and NAWM), with Cochran’s Q = 3.27 (df = 1, *p* = 0.0706), indicating non-significant variability. The I^2^ statistic was 69.4%, suggesting heterogeneity for a two-study model. The τ^2^ value was 0.822, reflecting relevant dispersion in the effect size estimates.

Chen et al. was not included in the quantitative synthesis due to the absence of numerical group-level data [98]. The study reported only qualitative assessments of lactate and bicarbonate signal presence in tumour tissue versus contralateral white matter, precluding SMD estimation. Nonetheless, the directionality of metabolic contrast in Chen et al. is consistent with the findings of the other two studies, supporting the robustness of the overall trend.

### 3.4. Secondary Findings—Hyperpolarized MRI and Contrast Enhanced MRI

All three studies addressed the spatial and diagnostic relationship between hMRI and conventional gadolinium-based contrast-enhanced MRI (CE-MRI). Although none of the investigations performed a full diagnostic accuracy comparison, the integration of CE-MRI provided important contextual and anatomical references for evaluating metabolic alterations detected via hMRI imaging.

Autry et al. explicitly compared k_PL values in gadolinium-enhancing and non-enhancing lesions [116]. Elevated pyruvate-to-lactate conversion was observed in both lesion types, with higher k_PL values noted in enhancing regions (e.g., 0.041 ± 0.009 s^−1^) compared to non-enhancing tumour areas (0.024 ± 0.001 s^−1^), suggesting that hMRI captures metabolic activity beyond what is visible on CE-MRI. This indicates that hMRI imaging may provide complementary information regarding tumour viability and extent.

Chen et al. did not conduct a direct quantitative comparison but acknowledged potential interference between gadolinium and hyperpolarized ^13^C signal acquisition [98]. To avoid T1-shortening effects on ^13^C signals, CE-MRI was performed only after hMRI, demonstrating awareness of the sensitivity of metabolic imaging to exogenous contrast agents. The absence of lactate signal in contralateral white matter, combined with CE-MRI localization, suggested metabolic specificity of hyperpolarized imaging.

Zaccagna et al. used contrast-enhanced T1-weighted images as anatomical references for placing regions of interest and for biopsy targeting [69]. Metabolic maps were overlaid on CE-MRI images, enabling visual comparison of lactate and bicarbonate distributions relative to contrast-enhancing regions. However, no statistical correlation or diagnostic performance analysis was conducted. Notably, heterogeneity in ^13^C signal intensity was observed both within and beyond areas of contrast enhancement.

Secondary findings highlighted the feasibility of integrating hMRI into clinical workflows. The included studies reported acceptable safety profiles and logistical considerations, such as the need for rapid sample preparation and specialized equipment.

## 4. Discussion

We conducted a systematic review on the application of hMRI in neurosurgery and identified three studies. The QUADAS-2 assessment revealed moderate methodological quality among the included studies. Key limitations identified include potential index test bias in certain studies and inconsistencies in the reference standards and flow and timing reports used. The studies generally exhibited moderate to high methodological quality. The data support a consistent tumour-to-control metabolic contrast observed across studies, albeit with moderate variability in magnitude. The limited number of included quantitative datasets necessitates cautious interpretation of heterogeneity statistics. Together, these findings suggest that hMRI provides functional metabolic information that may extend beyond the anatomical boundaries defined by conventional contrast enhancement. While CE-MRI remains essential for structural localization, hMRI holds potential for detecting tumour metabolism in both enhancing and non-enhancing regions, underscoring its relevance in surgical planning and response assessment.

### 4.1. Current Surgical and Technical Concepts for Primary Brain Tumours

The safety and efficacy of neurosurgical interventions depend heavily on the precision with which lesions—such as intrinsic brain tumours—are localized, characterized, and resected [117,118,119,120,121,122,123]. Techniques such as functional MRI (fMRI), diffusion tensor imaging (DTI), and iMRI have significantly enhanced the ability to visualize critical brain structures and assess tumour infiltration into functional areas [124,125,126,127,128,129,130]. For instance, iMRI has been shown to improve the extent of resection (EOR) and progression-free survival (PFS) in patients with gliomas, while minimizing the risk of postoperative deficits [8,11,15,16,17,18,26,31,62,119,131,132,133,134,135,136]. Additionally, the use of fluorescent agents such as 5-ALA has enabled surgeons to better delineate tumour margins during surgery, leading to higher rates of GTR and improved prognostic outcomes [22,119,137,138,139,140]. However, 5-ALA has the disadvantage that it cannot be used preoperatively to visualise the extent of the tumour, does not offer good spatial resolution for eloquent areas and does not show any connection to important fibre tracts. Intraoperative electrophysiology, including techniques such as direct cortical stimulation (DCS), continuous motor evoked potential (MEP) monitoring, and somatosensory evoked potentials (SSEPs), has further enhanced surgical precision by enabling real-time functional mapping [141,142,143]. These tools allow neurosurgeons to identify and preserve eloquent brain regions while maximizing tumour resection [123,144,145,146]. Mapping techniques, including navigated transcranial magnetic stimulation (nTMS) and DCS, have made previously inoperable tumours accessible, particularly in eloquent regions [118,120,147]. The integration of electrophysiological tools with advanced imaging techniques, such as iMRI or 5-ALA, has become crucial in order to allow a robust onco-functional balance, allowing surgeons to maximize tumour resection while safeguarding neurological and cognitive functions [118,120,148]. There is a synergistic effect of combining electrophysiology with 5-ALA fluorescence and intraoperative imaging, resulting in improved resection rates and neurological outcome [145,146,149,150]. Intraoperative ultrasound (iUS), proven as a cost-effective method, provides real-time feedback during surgery, particularly in low-grade gliomas [151]. It improves GTR rates and overall survival while reducing the risk of recurrence compared to conventional neuronavigation [7,22,151]. Combining iMRI, iUS, and 5-ALA have demonstrated superior outcomes compared to single-modality techniques [152,153,154]. Unfortunately, all methods have the disadvantage of not being able to fully visualise the biological growth of the primary brain tumour.

### 4.2. Imaging Diagnostics in Neurosurgery and Tumour Biology

The classification of intrinsic brain tumours has undergone several iterations of refinement by the WHO, moving from purely histological to increasingly molecular and genetic-based criteria [110,155,156,157]. The WHO classification versions now incorporate molecular parameters that can drastically influence prognosis, expected progression patterns, and responses to adjuvant therapies (radiation and chemotherapy) [110]. The genetic analyses often require days or weeks for completion [158,159,160,161]. As a result, adjuvant therapies, relying on molecular classifications, may be delayed [162]. This is particularly problematic in aggressive tumours where early intervention could influence outcomes. Furthermore, there is no direct intraoperative imaging modality currently available that can predict or correlate with the genetic and molecular classification of the tumour. Therefore, while GTR of contrast-enhancing tumour components correlates with better outcomes for some tumour types, this relationship can vary widely depending on the tumour’s underlying molecular profile [163,164]. Achieving a radiological GTR on MRI does not guarantee that the most biologically aggressive or metabolically active tumour cells have been removed [8,165,166,167,168]. Indeed, there may be a disconnect between the imaging-defined boundaries and the genetic or molecular composition that drives tumour behaviour [1,34,37,38,166,169,170]. Current intraoperative imaging techniques contribute little to resolving this disconnect [164,170,171]. They do not provide real-time insight into the tumour’s metabolic or molecular state. This shortcoming means that even the most meticulous resection may leave behind cells that are poised for rapid recurrence. Given this context, the next logical step in intraoperative imaging would be to visualize beyond the conventional contrast-enhancing borders. This would involve imaging techniques that can identify metabolic differences between tumour tissue and normal brain parenchyma, potentially offering a more accurate surrogate for the true extent of disease [69,97,98,172]. hMRI represents a promising frontier in this regard.

hMRI is unique because it does not rely on gadolinium or other conventional contrast agents that highlight vascular permeability [74]. Instead, it provides a metabolic fingerprint of the tissue [114,170,173,174,175,176,177,178,179,180,181,182,183,184]. By hyperpolarizing certain nuclear spins, hMRI allows the visualization of metabolic substrates and their downstream products [88,90,92,94]. Tumour cells often exhibit a distinct metabolic phenotype (e.g., increased lactate production), and hyperpolarized MRI could, in principle, visualize these metabolic differences intraoperatively [74,76,77,82,88,89,90,91,98,99]. In other words, if conventional MRI, iMRI, and 5-ALA represent incremental improvements in delineating tumour boundaries, hMRI may offer a qualitative leap. It has the potential to show the “true” extent of infiltration by identifying not just which areas take up contrast or fluoresce under specialized light, but which areas metabolically behave like tumour [78]. This could guide resections in a way that aligns with the underlying tumour biology and molecular classification. To date, clinical application of hMRI in neurosurgery remains limited, and significant technical and logistic hurdles must be overcome. However, its promise lies in bridging the gap between structural or contrast-based imaging and the molecular reality of tumour behaviour. By integrating metabolic imaging into the intraoperative environment, neurosurgeons could potentially achieve a more biologically meaningful GTR—one that aligns more closely with the true tumour margins and, consequently, may influence patient survival and quality of life more effectively than current techniques.

### 4.3. Hyperpolarized MRI in Neurosurgery

Hyperpolarized MRI is a groundbreaking imaging technique that dramatically increase the signal intensity of specific molecules [75,108]. Unlike conventional MRI, which relies on the intrinsic polarization of nuclei in a strong magnetic field (typically only a tiny fraction of nuclear spins are aligned, leading to relatively low signal), hyperpolarization methods temporarily manipulate the spin states of these nuclei, boosting their magnetic resonance signal by several orders of magnitude [75,185]. This enhancement allows for the detection and quantification of metabolic substrates and their conversion to products in real time, providing a direct window into cellular metabolism [185].

From a technical standpoint, hyperpolarization often involves substrates labelled with carbon-13 (^13^C), a stable isotope that can be integrated into various metabolic molecules—most notably pyruvate, a key intermediate in glycolysis [74,88,186,187,188]. In a specialized polarizer system, these ^13^C-labeled substrates are cooled to extremely low temperatures and exposed to microwave irradiation in a strong magnetic field. This process aligns a large proportion of the ^13^C nuclear spins.

Several hyperpolarization techniques have been developed to enhance the sensitivity of ^13^C-labeled pyruvate in magnetic resonance imaging (MRI), with Dynamic Nuclear Polarization (DNP) being the most widely used. DNP involves cooling the sample to cryogenic temperatures and transferring polarization from unpaired electron spins (typically from a radical) to the ^13^C nucleus under microwave irradiation [189]. Other techniques include Parahydrogen-Induced Polarization (PHIP), which uses the spin order of parahydrogen to transfer polarization, and Signal Amplification by Reversible Exchange (SABRE), a variant of PHIP that does not require chemical transformation of the substrate [190,191,192,193]. While DNP currently offers the highest polarization levels and is clinically validated, ongoing research continues to improve the efficiency, scalability, and biocompatibility of these alternative methods, which are expected to be more efficient in clinical use [190,191,192].

Once rapidly dissolved and injected intravenously into the patient, the hyperpolarized substrate retains its elevated polarization for a short time (on the order of tens of seconds to a few minutes), during which MRI acquisition can visualize and quantify the distribution and metabolism of the substrate.

Tumours exhibit altered metabolism (Warburg effect), that can be detected by hyperpolarized ^13^C MRI [95,96,97]. For example, hyperpolarized ^13^C-pyruvate can be administered intravenously, and its conversion to ^13^C-lactate can be imaged. Tumour regions with high metabolic activity produce more lactate signal, delineating areas of active tumour metabolism beyond what might be visible by conventional MRI contrast enhancement [84,114,174,178,179,181,182,184]. This approach fundamentally differs from gadolinium-based contrast enhancement (vascular and permeability differences versus intrinsic metabolic activity) [170].

A neurosurgeon working with hMRI could, in theory, pinpoint the true metabolic boundaries of a brain tumour by neuronavigation. Unlike contrast-based imaging, which fails to depict non-enhancing, infiltrative tumour margins, hMRI maps out regions of abnormal metabolism. Thus, the technique might identify microscopic disease infiltration long before it becomes visible through conventional imaging methods [166,194,195,196,197]. This can be concluded from the results of the studies reviewed. These metabolic boundaries could guide a more biologically meaningful resection, removing tissue that—while not contrast-enhancing—still harbours viable tumour cells.

From an operative standpoint, integrating hMRI into the neurosurgical workflow would be challenging but potentially transformative. A dedicated hyperpolarization unit would be required, and careful coordination would be needed to produce and deliver the hyperpolarized substrate in a timely manner. MRI-compatible neurosurgical environments, possibly akin to the advanced intraoperative MRI suites that already exist, would need to incorporate the hyperpolarization infrastructure. Surgeons would also need training in interpreting metabolic imaging data intraoperatively. Over time, neurosurgeons may move beyond resecting only the contrast-enhancing lesion to removing tumour along metabolic gradients. Such an approach would require careful balancing: more extensive resections carry a greater risk of neurological deficits, so functional mapping and electrophysiological monitoring would remain crucial.

### 4.4. Future Neurosurgical Hypotheses and Implications

Another important consideration is how hMRI could influence the understanding of the genetic and molecular basis of tumours. There is currently no direct way to observe WHO relevant metabolic differences intraoperatively [29,32,110,111,112,156]. If hMRI could detect differences in metabolic signatures that correspond to specific genetic subtypes, it might help stratify patients in real time and potentially guide intraoperative decisions. Although this is a future aspiration rather than a present reality, it aligns perfectly with the direction in which neuro-oncology is heading: an era of personalized, precision medicine [198,199].

In the context of tumour migration and infiltration, understanding metabolic boundaries could also influence adjuvant therapies. Radiation oncologists and medical oncologists frequently rely on postoperative imaging to define radiation fields or plan chemotherapy regimens [200,201,202,203,204]. If hyperpolarized MRI identifies a larger area of metabolically active disease, these treatments could be more accurately targeted, potentially improving their efficacy [72,98,205,206,207,208]. Moreover, if surgeons are able to achieve more complete metabolic resections, patients may have a lower burden of residual disease, potentially improving the effectiveness of adjuvant treatments and patient prognosis.

The current limitations of hyperpolarized MRI include the need for specialised equipment, the short lifetime of the hyperpolarized state and the need for rapid image acquisition and often limited spatial fidelity. In its current form, hMRI is unable to provide spatial resolution in the range of a few millimetres. This can limit the detection of very small tumour infiltrations. Current efforts are aimed at further developing hMRI as a hot-spot imaging technique without background signal, similar to PET, to achieve significantly better spatial representation.

Additionally, the technology is expensive and not yet widely available. Clinical use has, to date, been limited primarily to research settings and the interpretation of results requires experienced professionals. Expanding its availability would require collaboration between manufacturers, hospital systems, regulatory bodies, and research institutions. In addition, the evidence base is limited due to the small number and heterogeneity of studies. Furthermore, robust clinical trials would be needed to demonstrate that hMRI actually improves outcomes compared to conventional imaging and resection strategies. These factors highlight the need for further research to enable the wider application and implementation of this technology in neurosurgical neuro-oncology.

If metabolic imaging changes the definition of the tumour boundary, surgeons may need to consider resecting tissue that appears “normal” under conventional imaging but demonstrates abnormal metabolism. This practice would have profound implications, raising new questions about risk–benefit analyses, informed consent, and patient counselling. It could also spur innovation in intraoperative functional mapping, to ensure that removing these metabolically active but structurally “invisible” tumour regions does not compromise eloquent brain function. Finally, metabolic imaging might allow a more nuanced approach: maximal safe resection defined not only by anatomical imaging and function preservation but also by metabolic characterization. In other words, surgeons could aim to remove the metabolically active tumour core and its immediate infiltration zone, rather than relying on structural and permeability markers that may be less relevant to the tumour’s true biological aggressiveness.

### 4.5. Limitations of This Analysis

This systematic review has some limitations. The included studies showed substantial methodological heterogeneity. This limits the interpretability of the results and suggests that the studies available may not be sufficiently comparable for a robust quantitative synthesis. Heterogeneity prevented the performance of a statistical meta-analysis. In addition, many studies were pilot studies and analysed small patient samples, which limits the generalisability of the results. In addition, publication bias may have influenced the results, as studies with negative results are published less frequently. Finally, the quality of some of the included studies was affected by unclear bias. These limitations highlight the need for further, methodologically robust studies with larger patient cohorts and standardised protocols.

## 5. Conclusions

The current contrast-enhanced MRI technologies and the new WHO classifications are not able to replicate the true infiltrative nature of intrinsic brain tumours and the complexity of these tumours, highlighting genetic and molecular heterogeneity. However, the current intraoperative toolkit offers little guidance about these molecular realities, and the reliance on contrast enhancement for tumour delineation remains a fundamental limitation. Hyperpolarized MRI could represent a potential leap forward, offering a metabolic perspective on tumour biology that goes beyond the borders defined by contrast agents. By visualizing metabolic activity, hMRI may empower neurosurgeons to identify, delineate, and resect tumour tissue more accurately than ever before. This metabolic approach could better align surgical strategies with the biological and genetic underpinnings of the disease, thereby improving patient survival, guiding more targeted adjuvant therapies, and ultimately transforming patient care. For the neurosurgeon, the integration of hMRI could mean more confident decision-making intraoperatively. For the neuroradiologist, it provides a tool that bridges the gap between structural imaging and metabolic reality. For the oncologist and radiation therapist, it offers a more nuanced understanding of residual disease, enabling more precise adjuvant treatments. And most importantly, for the patient, it holds the promise of improved outcomes, both in terms of prolonged survival and better quality of life. hMRI adoption will require interdisciplinary collaboration, technical refinement, and careful clinical validation. Research must be advanced in the direction where metabolic imaging guides neurosurgical resection, is complementing genetic and molecular insights, and is helping to realize the full potential of precision medicine in the fight against devastating brain tumours.

## Figures and Tables

**Table 1:** Characteristics of the included studies (T = Tesla, HP = hyperpolarization, Normal Appearing White Matter = NAWM, Normal Appearing Brain Parenchyma = NABP).

Study	Mean Age (± SD)	Male/Female	Design	Population	Field Strength	HP Agent	Main Outcomes	Control Region
Autry et al. (2020) [116]	45.6 ± 10.0	2:3	Prospective observational	5 patients with glioma	3T MRI	^13^C pyruvate	kPL and kPB values	NAWM (normal-appearing white matter)
Chen et al. (2021) [98]	67.0 ± 4.2	2:1	Case series	3 patients with glioblastoma	3T MRI	^13^C pyruvate	Relative lactate and bicarbonate signals	Contralateral NAWM
Zaccagna et al. (2022) [69]	60.0 ± 10	6:2	Prospective observational	7 patients with glioma	3T MRI	^13^C pyruvate	Lactate/Bicarbonate ratio Lactate/Pyruvate ratio kPL and kPB values	Contralateral NABP

**Table 2:** Effect Size Summary (FLAIR T2-hyperintense lesion = T2L, Glioblastoma = GBM, Normal Appearing Brain Parenchyma = NABP, Normal Appearing White Matter = NAWM, Pyruvate-to-lactate ratio = kPL, Standardized Mean Difference = SMD).

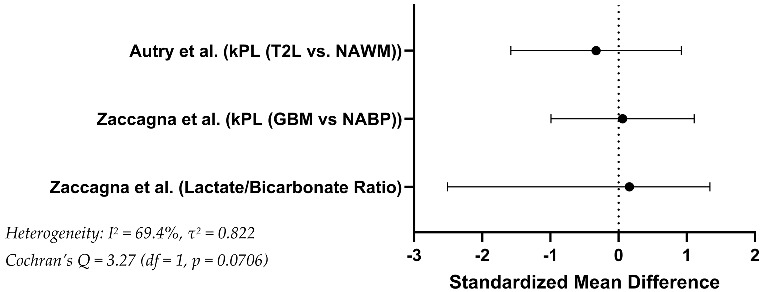										
Study	Outcome Variable	Group A (Mean ± SD) Non-Tumour Side	Group B (Mean ± SD) Tumour	Group Sizes (A/B)	Test Reported in Study	Statistical Method for SMD Computation	*p*-Value	SMD	Standard Error	95% CI
Zaccagna et al. [69]	Lactate/Bicarbonate Ratio	0.1043 ± 0.0412	0.0571 ± 0.0281	n = 7/n = 7	Wilcoxon test	Cohen’s d (pooled)	0.002	1.34	0.600	−2.51–0.16
	kPL (GBM vs. NABP)	16.51 ± 7.86	16.09 ± 6.18				0.730	0.06	0.535	−0.99–1.11
Autry et al. [116]	kPL (T2L vs. NAWM)	0.0198 ± 0.0055	0.0216 ± 0.0054	n = 5/n = 5	Not specified	Cohen’s d (pooled)	Not reported	−0.33	0.638	−1.58–0.92
Chen et al. [98]	Relative Lactate/Bicarbonate Signal	Not reported in analyzable format	Not reported in analyzable format	n = 3/n = 3	Qualitative only	Not applicable	Not applicable	Not calculable	Not calculable	Not calculable

## Data Availability

The original data presented in the study are openly available in the descripted medical literature databases. Further information can be given on request by the authors.

## Supplementary Materials

The following supporting information can be downloaded at: https://www.mdpi.com/article/10.3390/curroncol32110597/s1, Table S1: Results of QUADAS-2 Risk of Bias Assessment.

## Abbreviations

The following abbreviations are used in this manuscript:

5-ALA, ALA	5-aminolevulinic acid
BBB	blood–brain barrier
C	Carbon
CT	computed tomography
CE-MRI	contrast-enhanced MRI
kPL	conversion rates of pyruvate to lactate
kPB	conversion rates of pyruvate to bicarbonate
DESI-MS	desorption electrospray ionisation mass spectrometry
df	degrees of freedom
DTI	diffusion tensor imaging
DCS	direct cortical stimulation
EOR	extent of resection
fMRI	functional MRI
GTR	gross total resection
hMRI	hyperpolarized magnetic resonance imaging
iUS	intraoperative ultrasound
iMRI	intraoperative MRI
MR	magnet resonance
MRI	magnetic resonance imaging
MRSI	magnetic resonance spectroscopic imaging
MEP	motor evoked potential
nTMS	navigated transcranial magnetic stimulation
NABP	normal appearing brain parenchyma
NAWM	normal-appearing white matter
PET	positron emission tomography imaging
PFS	progression-free survival
QUADAS-2	Quality Assessment of Diagnostic Accuracy Studies
SSEPs	somatosensory evoked potentials
SMD	standardized mean differences
TCA	tricarboxylic acid
WHO	World Health Organization
