# Supplementary material for: Metabolic Imaging as Future Technology and Innovation in Brain-Tumour Surgery: A Systematic Review
Source: Curr Oncol. 2025 Oct 24;32(11):597. doi: 10.3390/curroncol32110597 (PMC12651585; doi:10.3390/curroncol32110597)
Supplement: Supplementary file 1 [file curroncol-32-00597-s001.zip › Supplement 1 QUADAS Assessment.pdf]

Supplement Table S1: QUADAS-2 Risk of Bias Assessment

Study	Patient Selection (Bias)	Justification – Patient Selection	Index Test (Bias)	Justification – Index Test	Reference Standard (Bias)	Justification – Reference Standard	Flow & Timing (Bias)	Justification – Flow & Timing
Autry et al. (2020)	LOW	Prospective recruitment; no selection bias; no inappropriate exclusions.	LOW	Well-described standardized index test; no indication of diagnostic review bias.	UNCLEAR	No formal reference standard like histology applied to validate metabolic readings.	LOW	Complete patient flow; clearly documented time intervals; no dropouts.
Chen et al. (2021)	LOW	Three consecutively enrolled patients with suspected GBM; no case-control design.	UNCLEAR	Interpretation blinding not explicitly described; potential for observer bias.	LOW	Histopathological confirmation used consistently across all patients.	UNCLEAR	Timing (1–2 days before surgery) is plausible, but reference standard application consistency unclear.
Zaccagna et al. (2022)	LOW	Consecutive patients with defined inclusion and exclusion criteria.	UNCLEAR	Blinding of index test interpretation not explicitly stated.	UNCLEAR	Reference standard used (pathology) but blinding procedures unclear.	LOW	Short time interval between imaging and surgery; all included except 1 dropout.
